# The association between smoking behaviors and prices and taxes per cigarette pack in the United States from 2000 through 2019

**DOI:** 10.1186/s12889-022-13242-5

**Published:** 2022-04-28

**Authors:** Thuy T. T. Le, Mohammed A. Jaffri

**Affiliations:** 1 Department of Health Management and Policy, School of Public Health, University of Michigan, Ann Arbor, MI USA; 2 Department of Epidemiology, School of Public Health, University of Michigan, Ann Arbor, MI USA

## Abstract

**Objective:**

The conclusions on how tax and price increases affect smoking behaviors are mixed. This work is devoted to re-evaluating the relationship between cigarette prices and taxes and smoking behaviors.

**Methods:**

Using 2000–2019 Behavioral Risk Factor Surveillance System data, we employed linear mixed-effect models to re-examine the impact of cigarette prices and taxes on smoking prevalence and the proportion of current smokers having tried to quit smoking in the past 12 months. All the analyses were conducted for the general population, then by age group, gender, race/ethnicity, and income level.

**Results:**

The results indicate that higher cigarette prices and taxes were associated with a decrease in smoking prevalence and an increased likelihood of quitting smoking. Cigarette tax and price increases produced the most powerful impact on the smoking prevalence of 18- to 24-year-olds. The estimates also show that males tended to be more price-sensitive than females. Raising cigarette prices and taxes was estimated to be more effective in reducing the smoking prevalence among non-Hispanic Blacks and Hispanics when compared to non-Hispanic whites. Cigarette price and tax changes were likely to have a smaller effect on individuals with annual income under $25,000 relative to individuals with higher income levels.

**Conclusions:**

Increases in cigarette prices and taxes are significantly associated with a reduction in smoking prevalence and an increased likelihood of quitting smoking among adults across different demographic and socioeconomic groups. However, as cigarette price and tax changes disproportionately affect low-income individuals, raising cigarette prices and taxes may deepen income disparities.

## Introduction

Cigarette smoking is the leading preventable cause of death in the United States. Cigarette smoking, a serious public health issue, claims about 480,000 lives annually [1]. Many tobacco control interventions, such as cigarette taxes, warning labels, smoke-free indoor air laws, and public health messages, have been introduced to discourage smoking initiation and encourage smoking cessation [1, 2]. Among all interventions, increasing cigarette taxes, and thus increasing the price per cigarette pack, is documented to be the most effective strategy in lowering smoking prevalence, especially among young people and people with low socioeconomic status [1, 3, 4].

There are a growing number of studies in the literature investigating the relationship between smoking behaviors and cigarette prices and taxes among adults [4–12] and adolescents [13–15]. Some studies have found that cigarette tax and price increases have significant impacts on reducing smoking prevalence [5, 12, 16, 17]. Others have reported weak or no statistically significant effects of cigarette prices and taxes on cigarette use [18]. The evidence about how the effects of tax and price increases are distributed across subpopulations is also inconsistent. For instance, previous work [19] concluded that increased taxes and prices work more effectively among low-income individuals, while a recent study [5] suggested the contrary. A comprehensive understanding of the impacts of cigarette taxes and prices is essential for policymakers. Thus, these mixed conclusions require more research on this topic.

Inspired by the work of Sharbaugh et al. [5] and to add to the existing conversation, we used Behavioral Risk Factor Surveillance System (BRFSS) data from 2000 through 2019 to comprehensively investigate the effects of cigarette taxation and pricing per pack of 20 cigarettes on smoking behaviors. The analyses are performed for the general population, then by age, gender, race/ethnicity, and income. Our analysis builds on previous work [5] in three ways. First, we use a longer and more contemporary period, from 2000 through 2019 (from 2001 through 2014 in [5]). Extending the analysis period to the most recent years is essential given the rapidly changing landscape of tobacco use during the past several years. Second, we include more detailed measures of cigarette tax and price. In particular, we employ the federal and state taxes per pack (in dollars), the federal and state taxes as the percentages of the average retail prices and the average retail prices as the measures for cigarette taxation and pricing, and smoking prevalence and the prevalence of quit attempts for smoking behaviors. Third, our results are explicitly adjusted for state tobacco control programs, state smoke-free indoor air laws, autocorrelation, inflation, and the changes in the BRFSS methodology in 2011, making them more reliable than previous work that did not include these adjustments [5].

## Methods

### State-specific smoking prevalence and the prevalence of quit attempts

The BRFSS is a state-based system of health-related telephone surveys that collect information on health-related risk behaviors, chronic health conditions, and the use of preventative services in all 50 states, the District of Columbia, and three US territories [20]. Originally, the BRFSS surveys were conducted via landline phone. As the use of cell phones increased, the surveys were conducted via cell phone to produce more representative data. As a result, after several years of trial, the 2011 BRFSS data set includes cell phone survey data in addition to the landline phone survey data. Furthermore, in 2011, the post-stratification weighting method, which was used for the previous BRFSS data, was replaced by a new weighting methodology, raking. These changes cause trendline breaks and prevent direct comparison of BRFSS estimates after 2011 to those before 2011 [21]. To reflect the 2011 methodological changes, a dummy variable was included in the model as described below.

The smoking prevalence and the prevalence of quit attempts (as the proportion of current smokers who have made at least one quit attempt in the past 12 months among current smokers) for each of 50 states and the District of Columbia were extracted from the 2000–2019 BRFSS data. Here, current smokers are defined as adult smokers who have smoked at least 100 cigarettes in their lifetime and currently smoke on at least some days. Additionally, quit attempts are defined as stopping smoking in the past 12 months with the intention of quitting.

### State-specific cigarette taxes and prices

The data on federal and state cigarette taxes (in dollars and as the percentages of the average retail prices) and the average retail prices for each of the 50 states and the District of Columbia from 2000 through 2019 were taken from the Tax Burden on Tobacco by Orzechowski and Walker [22]. To accurately estimate the effectiveness of cigarette taxation and pricing in reducing smoking behaviors, the cigarette taxes and prices in dollars were converted to the 2019 dollar value using the Consumer Price Index Research Series (CPI-U-RS) [23] over the same period.

### State tobacco control programs and comprehensive smoke-free laws

Centers for Disease Control and Prevention (CDC) first provided recommended funding ranges for state tobacco control programs to effectively reduce the numbers of new smokers, encourage and aid smokers to quit in 1999 [24] and subsequently updated in 2007 and 2014 [25, 26]. In this work, we used state tobacco control expenditures, e.g., percentages of CDC-recommended levels, as measures of whether or not states have spent enough money to reduce tobacco use [27]. A state can be considered as having comprehensive smoke-free laws if smoking is prohibited in all three venues, i.e., bars, worksites, and restaurants [28, 29]. Since state tobacco control programs and comprehensive smoke-free laws have been shown to affect the smoking prevalence and the prevalence of quit attempts [26], our estimates were adjusted for these factors to tease out the impacts of cigarette prices and taxes on smoking behaviors. The data on state tobacco control program funding and the status of state comprehensive smoke-free air laws were taken from [27] and [28–30] respectively.

### Statistical analysis

Due to the nature of the state-specific longitudinal data used in this study, we utilized linear mixed-effect models with temporary autocorrelation to investigate the association between cigarette taxes and prices and smoking behaviors while taking into account the heterogeneity across the 50 states and the District of Columbia. The smoking prevalence and the prevalence of quit attempts were considered as dependent variables, while the federal and state cigarette taxes (in dollars and as the percentages of the average retail prices) and average cigarette prices per pack were the key independent variables. Furthermore, a dummy variable was also added to incorporate the changes in the BRFSS weighting and survey methodologies. The estimates were adjusted for state tobacco control programs, state smoke-free air laws, and temporal autocorrelation using an autoregressive–moving-average model [31] (ARMA(2,1)). We first carried out the analysis for the general population and then for sub-populations stratified by age group, gender, race, and income to examine the effectiveness of cigarette pricing and taxation across different demographic and socioeconomic groups.

In Table 1, the prevalence of cigarette smoking was the mutual dependent variable in all three models, while various measures of cigarette price and tax were subsequently employed as the main independent variable in Models 1–3 (i.e., average retail prices in Model 1, federal and state taxes per pack (in dollars) in Model 2, and federal and state taxes per pack (as percentages of average retail prices) in Model 3). Similarly, in Table 2, the prevalence of quit attempts is the only dependent variable for all three models. In addition, the sensitivity analyses were performed to test the robustness of the models by restricting the data to the period from 2000 through 2010.

**Table 1:** The association between smoking prevalence and cigarette prices and taxes (average retail prices in Model 1, federal and state taxes per pack (in dollars) in Model 2, and federal and state taxes per pack (as percentages of average retail prices) in Model 3)

	**Model 1** (Average retail prices ($))			**Model 2** (Federal and state taxes per pack ($))			**Model 3** (Federal and state taxes per pack as percentages of average retail prices (%))		
	**Value**	**95% CI**	***p*-value**	**Value**	**95% CI**	***p*-value**	**Value**	**95% CI**	***p*-value**
**Total**	-0.52	(-0.71, -0.33)	< 0.001	-0.65	(-0.88, -0.42)	< 0.001	-0.05	(-0.07, -0.03)	< 0.001
**By age groups**									
18–24	-1.80	(-2.3, -1.29)	< 0.001	-1.87	(-2.49, -1.25)	< 0.001	-0.15	(-0.22, -0.09)	< 0.001
25–49	-0.73	(-0.99, -0.48)	< 0.001	-0.92	(-1.23, -0.62)	< 0.001	-0.08	(-0.11, -0.05)	< 0.001
50 +	-0.43	(-0.58, -0.28)	< 0.001	-0.56	(-0.75, -0.38)	< 0.001	-0.05	(-0.07, -0.03)	< 0.001
**By gender**									
Males	-0.77	(-1.03, -0.52)	< 0.001	-0.95	(-1.26, -0.65)	< 0.001	-0.08	(-0.11, -0.05)	< 0.001
Females	-0.59	(-0.78, -0.39)	< 0.001	-0.73	(-0.96, -0.5)	< 0.001	-0.06	(-0.09, -0.04)	< 0.001
**By race**									
NH White	-0.52	(-0.72, -0.32)	< 0.001	-0.71	(-0.96, -0.47)	< 0.001	-0.06	(-0.09, -0.04)	< 0.001
NH Black	-1.48	(-2.13, -0.83)	< 0.001	-1.79	(-2.58, -1.00)	< 0.001	-0.16	(-0.24, -0.08)	0.001
Hispanic	-1.30	(-1.83, -0.76)	< 0.001	-1.50	(-2.15, -0.86)	< 0.001	-0.15	(-0.21, -0.08)	< 0.001
Other	-1.34	(-1.82, -0.86)	< 0.001	-1.47	(-2.05, -0.90)	< 0.001	-0.12	(-0.18, -0.07)	< 0.001
**By income**									
0 K—< 25 K	-0.39	(-0.68, -0.11)	0.01	-0.42	(-0.76, -0.07)	0.02	-0.03	(-0.06, 0.00)	0.08
25 K—< 50 k	-0.90	(-1.16, -0.65)	< 0.001	-1.14	(-1.45, -0.84)	< 0.001	-0.11	(-0.14, -0.08)	< 0.001
50 K—< 75 K	-1.02	(-1.27, -0.77)	< 0.001	-1.30	(-1.60, -1.01)	< 0.001	-0.12	(-0.15, -0.09)	< 0.001
75 K +	-0.90	(-1.10, -0.69)	< 0.001	-1.14	(-1.38, -0.89)	< 0.001	-0.11	(-0.14, -0.09)	< 0.001

**Table 2:** The association between the prevalence of quit attempts and cigarette prices and taxes (average retail prices in Model 1, federal and state taxes per pack (in dollars) in Model 2, and federal and state taxes per pack (as percentages of average retail prices) in Model 3)

	**Model 1** (Average retail prices ($))			**Model 2** (Federal and state taxes per pack ($))			**Model 3** (Federal and state taxes per pack as percentages of average retail prices (%))		
	**Value**	**95% CI**	***p*-value**	**Value**	**95% CI**	***p*-value**	**Value**	**95% CI**	***p*-value**
**Total**	1.28	(0.95, 1.61)	< 0.001	1.71	(1.32, 2.11)	< 0.001	0.21	(0.17, 0.25)	< 0.001
**By age groups**									
18–24	0.63	(0.14, 1.12)	0.01	0.84	(0.22, 1.46)	0.01	0.07	(0.01, 0.14)	0.03
25–49	1.45	(1.05, 1.85)	< 0.001	1.96	(1.48, 2.45)	< 0.001	0.25	(0.20, 0.29)	< 0.001
50 +	1.77	(1.35, 2.19)	< 0.001	2.32	(1.83, 2.82)	< 0.001	0.30	(0.26, 0.34)	< 0.001
**By gender**									
Males	1.27	(0.89, 1.66)	< 0.001	1.83	(1.37, 2.28)	< 0.001	0.23	(0.18, 0.27)	< 0.001
Females	1.29	(0.92, 1.67)	< 0.001	1.67	(1.21, 2.12)	< 0.001	0.20	(0.16, 0.25)	< 0.001
**By race**									
NH White	1.22	(0.90, 1.54)	< 0.001	1.66	(1.27, 2.05)	< 0.001	0.20	(0.16, 0.23)	< 0.001
NH Black	0.53	(-0.48, 1.53)	0.31	0.77	(-0.51, 2.05)	0.24	0.08	(-0.05, 0.22)	0.23
Hispanic	1.33	(0.52, 2.13)	< 0.001	1.74	(0.75, 2.73)	< 0.001	0.19	(0.09, 0.30)	< 0.001
Other	1.36	(0.76, 1.96)	< 0.001	2.02	(1.27, 2.77)	< 0.001	0.25	(0.17, 0.33)	< 0.001
**By income**									
0 K—< 25 K	1.45	(1.02, 1.87)	< 0.001	1.95	(1.45, 2.45)	< 0.001	0.23	(0.19, 0.28)	< 0.001
25 K—< 50 k	1.54	(1.09, 1.99)	< 0.001	2.02	(1.47, 2.57)	< 0.001	0.23	(0.18, 0.29)	< 0.001
50 K—< 75 K	1.22	(0.75, 1.69)	< 0.001	1.87	(1.30, 2.44)	< 0.001	0.25	(0.19, 0.31)	< 0.001
75 K +	1.39	(0.92, 1.86)	< 0.001	1.93	(1.34, 2.51)	< 0.001	0.22	(0.15, 0.28)	< 0.001

All the analyses were performed using R’s “lme” function in the “nlme” package. All methods were performed in accordance with the relevant guidelines and regulations.

## Results

### Effects of cigarette prices and taxes on smoking prevalence

Table 1 shows the estimates of the positive impacts of cigarette price and tax on smoking prevalence across different groups. The effects of cigarette prices and taxes on smoking prevalence are qualitatively consistent across all three models. In particular, all the estimates in models 1–3 show that increases in cigarette prices and taxes lead to the declines in the prevalence of cigarette smoking across all demographic and socioeconomic groups (regardless of whether the average retail prices, the federal and state taxes per pack (in dollars), or the federal and state taxes per pack as percentages of average retail prices was used as the independent variable) over the 2000–2019 period. For instance, row 1 in Table 1 indicates that a $1 increase in the average retail prices in Model 1 (the federal and state taxes per pack in Model 2) corresponds with a decrease of 0.52 percentage points (0.65 percentage points in Model 2) in the population smoking prevalence. Meanwhile, in Model 3, if the federal and state taxes per pack as the percentages of average retail prices go up by 1%, the population smoking prevalence drops by 0.05 percentage points. The results of the three models consistently show that among all age groups, 18- to 24-year-olds are the most price-sensitive. Males tend to be more reactive to price changes than females. Non-Hispanic Whites and individuals whose income is less than $25,000 per year are the least sensitive in comparison with the remaining racial groups and income levels.

### Effects of cigarette prices and taxes on the prevalence of quit attempts

Table 2 shows the estimates of the positive impacts of cigarette price and tax on the prevalence of quit attempts across different groups. Here, the prevalence of quit attempts is the only dependent variable for all three models. The results show a consistently inverse association between cigarette prices and taxes and the proportion of smokers who have attempted to quit smoking in the past 12 months in all models, and across demographic and socioeconomic groups. Like Table 1, the first row in Table 2 presents that an additional $1 increase in the average retail prices in Model 1 (in the federal and state taxes per pack in Model 2) is associated with an increase of 1.28 percentage points (1.71 percentage points in Model 2) in the proportion of smokers trying to quit smoking in the past 12 months in the general population. Furthermore, increasing the federal and state taxes per pack as the percentages of the average retail prices by 1 percentage point raises the prevalence of quit attempts by 0.21 percentage points. The positive impact of cigarette price and tax on quit attempts is least significant among 18- to 24-year-olds, relative to other age groups, and seems to be muted among Non-Hispanic Blacks. Furthermore, when stratifying by gender and income, the increased likelihood of making quit attempts due to cigarette price and tax raises are not statistically different from each other.

The sensitivity analyses conducted by restricting the data to the period from 2000 through 2010 reconfirmed the robustness of the findings.

## Discussion

This work is devoted to re-evaluating the association between cigarette prices and taxes and smoking behaviors in the United States using BRFSS data from the 2000–2019 period. The estimates in Tables 1 and 2 reconfirm that higher cigarette prices and taxes significantly impact smoking prevalence and the proportion of smokers who make quit attempts [5, 16]. The results are qualitatively compatible across all three models. Our findings largely align with those reported by Sharbaugh et al. [5] with exceptions highlighted and discussed below.

Changes in cigarette taxes and prices produce the most powerful impact on 18- to 24-year-olds [3, 5]. Young adults are more sensitive to price changes than are other age groups, which is probably due to their income instability and the likelihood of having a less established smoking habit [19, 32]. One interesting fact is that, in this age group, the reduction in smoking prevalence (Table 1) is much more pronounced than the increase in the proportion of smokers who have made quit attempts in the past 12 months (Table 2), relative to the other age groups. Previous studies have pointed out that most smokers initiate smoking at early ages [33, 34]. Therefore, raising the cost of cigarettes is likely to primarily reduce the number of new established smokers, in addition to encouraging smoking cessation, which shapes the future smoking trend.

When stratifying by gender, our estimates indicate that males tend to be more price responsive than females [3], i.e., a 1-dollar increase in a cigarette price per pack lowers the smoking prevalence by 0.77 percentage points among males and 0.59 percentage points among females as estimated by Model 1 (Table 1). However, male and female smokers are likely to attempt to quit in response to the changes at the same rate as shown in Table 2. On the other hand, Sharbaugh et al. [5] reported no statistically significant difference in the cigarette price and tax effects on smoking prevalence among males and females.

According to the results from models 1–3, raising cigarette prices and taxes is estimated to be the most effective in reducing smoking consumption among non-Hispanic Blacks and Hispanics compared to non-Hispanic whites. The same observation has been reported in previous studies [3, 35]. However, this observation, as well as the one by gender (discussed above), is inconsistent with the findings in [5] based on the same dataset, BRFSS, but during different periods. These disagreements may stem from different factors. First, we used the BRFSS data from 2000 through 2019, while Sharbaugh et al. did from 2001 through 2014. Second, in contrast to Sharbaugh et al. [5], our results were explicitly adjusted for state tobacco control programs, smoke-free laws, temporal autocorrelation, inflation, and the changes in weighting and surveying methodologies that occurred in 2011, which makes our estimates more reliable. Table 2 shows Non-Hispanic Blacks are least likely to make quit attempts in the past 12 months in response to the increases in cigarette prices and taxes across all races.

Cigarette price and tax changes seem to have a minimal effect on individuals with annual income under $25,000, relative to higher income levels [5, 36]. In particular, raising the cigarette prices per pack by one dollar is associated with a decline of 0.39 percentage points in the smoking prevalence among participants with annual income below $25,000, whereas the decline is more than 0.90 percentage points among those with an annual income greater than $25,000 (Model 1 in Table 1). This is not a surprise since low-income individuals have been found to have a high smoking prevalence [37]. Even though the likelihood of having made a quit attempt in the past 12 months is similar across income groups (Table 2), the lack of a significant decline in smoking prevalence among the lower-income group is probably due to their limited exposure to cessation treatment and anti-smoking programs. Poor working conditions are also linked to high smoking prevalence. Both low- and high-income smokers pay the same tax amount per cigarette pack, so low-income smokers spend a disproportionately large share of their income on smoking. Thus, if low-income individuals are price-insensitive, as our study suggests, raising cigarette taxes and prices would further deepen income disparities.

Cigarette price and tax increases remain effective in lowering the smoking prevalence. However, these effects are heterogeneous across demographic and socioeconomic groups. Since there is no one-size-fits-all strategy, a combination of interventions should be considered to effectively reduce the smoking prevalence without further widening socioeconomic gaps. During the past decades, the smoking prevalence in the U.S. has decreased dramatically (from 23% in 2000 to 14% in 2019 based on the National Health Interview Surveys). This public health achievement is obviously not the result of the cigarette price and tax increases alone, but the combined effects of all tobacco control programs, policies, legislation, and advocacy such as tobacco prevention and cessation funding, smoking-free air laws, access to the cessation services at the federal, state and local levels [38]. In light of the complex and rapidly changing landscape of tobacco use, the popularity of new tobacco products, smoking behaviors need to be closely monitored to timely inform tobacco regulatory actions to protect public health.

Our study has some limitations. We used the state-specific BRFSS data from 2000 through 2019. We accounted for temporal autocorrelation; we did not account for spatial autocorrelation, while the smoking prevalence of neighboring states has a high chance of correlation. In addition, we considered only states with comprehensive smoke-free laws, while states with some levels of indoor smoking restriction may contribute to declines in smoking prevalence and quit attempts. There are probably other factors linked with positive changes in smoking behaviors that were not included in our models.

## Data Availability

The datasets used and/or analysed during the current study are available from the corresponding author on reasonable request.
